# Nutritional Value of Meat and Meat Products and Their Role in Human Health—2nd Edition

**DOI:** 10.3390/nu18010137

**Published:** 2025-12-31

**Authors:** Joanna Stadnik

**Affiliations:** Department of Animal Food Technology, Faculty of Food Science and Biotechnology, University of Life Sciences in Lublin, Skromna 8, 20-704 Lublin, Poland; joanna.stadnik@up.edu.pl

Meat and meat products have long been integral components of human diets, providing high-quality protein, essential amino acids, and a wide range of highly bioavailable micronutrients. Despite their nutritional value, meat consumption remains the subject of ongoing scientific and public debate due to concerns related to chronic disease risk, food processing, sustainability, and changing consumer attitudes.

Meat represents a nutrient-dense food matrix that delivers high-quality protein and several essential micronutrients in highly bioavailable forms. The protein fraction of meat is characterized by a complete indispensable amino acid profile and high digestibility, properties that support muscle protein synthesis and the maintenance of lean body mass, particularly in older adults and individuals with increased physiological protein requirements [1]. Nevertheless, much of the evidence underpinning these benefits is derived from short-term metabolic or feeding studies, limiting inference regarding long-term functional outcomes such as sarcopenia progression or morbidity.

A central nutritional contribution of meat concerns iron provision. Red meat is a major dietary source of heme iron, which is absorbed via distinct intestinal mechanisms and demonstrates substantially higher bioavailability than non-heme iron [2]. Evidence accumulated since 2015 indicates that heme iron intake plays a disproportionate role in determining iron status, particularly among women of reproductive age and individuals with marginal iron stores [3]. While recent intervention-based meta-analyses confirm that increased red meat consumption improves iron biomarkers, including serum ferritin and hemoglobin concentrations [4], heterogeneity in study design, baseline iron status, and intervention duration remains substantial. Moreover, few studies have assessed potential upper thresholds beyond which additional heme iron intake confers diminishing benefit or increased risk.

Meat is also a principal dietary source of vitamin B12, a micronutrient essential for neurological function and erythropoiesis. Observational data consistently demonstrate lower vitamin B12 status among individuals consuming diets that exclude animal-source foods [1]. However, reliance on serum B12 alone, rather than functional biomarkers such as methylmalonic acid, limits the precision of deficiency assessment in many studies. Similarly, although meat provides zinc and selenium in bioavailable forms, population-level intake data often fail to capture bioaccessibility or interactions with other dietary components [1,5].

The nutritional implications of meat processing represent a further area of complexity. While certain processing and preservation technologies may improve digestibility and food safety, industrially processed meats are frequently characterized by elevated sodium and nitrite levels, which are associated with adverse cardiometabolic and cancer-related outcomes [6]. Current evidence is predominantly epidemiological, underscoring the need for controlled intervention studies that disentangle the effects of processing methods from overall dietary patterns. Emerging reformulation strategies show promise in mitigating these risks, yet robust evidence regarding their long-term health impact remains limited [5,7].

In summary, while the nutritional value of meat is well supported, future research should prioritize long-term, well-controlled intervention studies, refined biomarker assessment, and systematic evaluation of processing innovations to better balance nutritional benefits against potential health risks.

The second edition of the Special Issue “Nutritional Value of Meat and Meat Products and Their Role in Human Health” was conceived to address these complex issues by presenting contemporary evidence that integrates nutritional composition, health outcomes, technological innovation, and consumer perspectives [Contributions 1–14].

Seventeen manuscripts were submitted for consideration for the Special Issue, and all of them were subject to the rigorous *Nutrients* review process. In total, fourteen papers (twelve research papers and two reviews) were finally accepted for publication and inclusion in this Special Issue.

The articles included in this Special Issue collectively demonstrate that the health effects of meat consumption cannot be evaluated in isolation. Instead, they depend on multiple interacting factors, including meat type, degree of processing, dietary context, and population-specific characteristics [Contributions 1–14]. This editorial summarizes the main contributions of the Special Issue and highlights key directions for future research.

This Special Issue includes original research articles providing updated and detailed data on the nutritional composition of meat and meat products. In particular, analyses of USDA Prime beef cuts demonstrate that, despite higher marbling, these products remain rich sources of protein, vitamin B12, niacin, zinc, selenium, and phosphorus, with relatively stable cholesterol content across cuts and cooking states [Contribution 6]. These findings are especially relevant in the context of changes in beef quality over recent decades and the need for accurate, up-to-date food composition databases.

Complementary evidence is provided by an original nutrient analysis of raw beef offal items published in this Special Issue, demonstrating that organs such as liver, heart, kidney, and tongue are nutrient-dense foods that can meaningfully contribute to intakes of essential vitamins and minerals [Contribution 5]. Together with a narrative review included in this Special Issue that highlights edible offal as a valuable yet underutilized dietary component, these articles emphasize the nutritional relevance of less commonly consumed meat products [Contribution 9].

In addition, the role of meat lipids is addressed in a comprehensive review published herein, which balances the nutritional benefits of meat lipids with potential health risks [Contribution 11]. This review underscores the importance of fatty acid composition, bioactive lipid compounds, and lipid oxidation, while also highlighting opportunities to improve lipid quality through animal feeding strategies, processing methods, and innovative product formulations.

A central theme addressed by studies published in this Special Issue is the evaluation of meat intake within the context of overall dietary patterns. Analyses of nationally representative NHANES data demonstrate that beef consumption is associated with higher intakes and improved adequacy of key nutrients commonly under-consumed in older adults, including protein, zinc, vitamin B12, and choline [Contribution 2]. Similar findings are reported among pregnant and lactating women, a population with increased nutritional requirements, further highlighting the contribution of beef to nutrient adequacy during critical life stages [Contribution 1].

Evidence from a randomized crossover controlled feeding trial included in this Special Issue demonstrates that healthy dietary patterns improve cardiometabolic risk factors regardless of whether they include lean, unprocessed beef or are entirely vegetarian [Contribution 7]. These findings emphasize that overall diet quality may be more important for cardiometabolic health than the exclusion of meat per se.

At the same time, epidemiological studies published herein highlight the importance of distinguishing between meat types and dietary context. Results from a screening colonoscopy population show that processed meat intake, rather than red meat alone, is associated with a higher prevalence of colorectal neoplasms, particularly when combined with genetic susceptibility [Contribution 8]. Similarly, data from the MICOL cohort indicate that the mortality risk associated with red meat consumption in individuals with metabolic dysfunction-associated steatotic liver disease is attenuated when diets include a higher proportion of leafy vegetables [Contribution 3]. Additional evidence from the EPIC-Spain cohort further elucidates potential mechanisms linking processed meat intake to cancer risk, focusing on nitrosyl-heme and heme iron exposure [Contribution 10].

This Special Issue also includes studies examining how meat processing and reformulation may influence health outcomes. A randomized clinical trial evaluating additive- and allergen-free reformulated cooked meat products suggests potential benefits related to inflammation, oxidative stress, gut microbiota composition, and perceived satiety, while also aligning with consumer demand for cleaner-label products [Contribution 12]. These findings highlight the potential for technological innovation to improve the health profile of processed meat products.

A long-term preclinical study published in this Special Issue demonstrates that dietary protein source and protein modification may exert sex-dependent effects on longevity, body composition, and metabolic outcomes [Contribution 4]. This work underscores that protein source and processing may influence health beyond total protein intake alone.

Consumer attitudes toward meat and alternative protein sources are explicitly addressed by studies included in this Special Issue. A cross-sectional study conducted in the United Arab Emirates reveals strong cultural attachment to conventional meat alongside cautious interest in cultured meat alternatives, with acceptance influenced by age, education, and sociocultural factors [Contribution 13]. These findings suggest that consumer education and cultural context will play a central role in shaping future dietary transitions.

In parallel, a comparative study of iron bioavailability published herein demonstrates that beef remains a highly bioavailable source of dietary iron compared with plant-based meat alternatives and insect-based products, although appropriately processed alternatives may still contribute to iron intake [Contribution 14]. This evidence highlights both the nutritional strengths of meat and the challenges faced by alternative protein sources in replicating its micronutrient bioavailability.

Collectively, the fourteen articles in this Special Issue advance a nuanced understanding of meat and meat products in human nutrition. The evidence highlights that meat can contribute meaningfully to nutrient adequacy and health when consumed in appropriate forms and dietary contexts, while also identifying areas where processing, excess intake, and product formulation warrant careful consideration.

Future research should prioritize dietary pattern-based approaches, improved characterization of meat components and processing-related compounds, and rigorous evaluation of reformulated and novel meat products in long-term human studies. Comparative research on alternative proteins and consumer acceptance will also be essential as food systems evolve.

As a Guest Editor, I hope that this Special Issue serves as a valuable reference for researchers, clinicians, and policymakers and stimulates further interdisciplinary research aimed at developing balanced, evidence-based guidance on meat consumption that integrates nutritional adequacy, health outcomes, and sustainability.

Considering the success of this Special Issue, we are pleased to announce that we are launching a third Special Issue on this topic entitled “Nutritional Value of Meat and Meat Products and Their Role in Human Health—3rd Edition”. Original research papers and reviews on topics presenting current knowledge on the nutritional value and health effects of meat and meat products are invited. We believe that this Special Issue will broaden the horizons of our knowledge on the role of meat and meat products in human health.
